# Effects of Zn, macronutrients, and their interactions through foliar applications on winter wheat grain nutritional quality

**DOI:** 10.1371/journal.pone.0181276

**Published:** 2017-07-26

**Authors:** Shaoxia Wang, Meng Li, Ke Liu, Xiaohong Tian, Shuo Li, Yanlong Chen, Zhou Jia

**Affiliations:** 1 College of Natural Resources and Environment, Northwest A&F University, Yangling, Shaanxi, China; 2 College of Hydraulic & Environmental Engineering, China Three Gorges University, Yichang, Hubei, China; 3 Engineering Research Center of Eco-Environment in Three Gorges Reservoir Region and Ministry of Education, China Three Gorges University, Yichang, Hubei, China; University of Delhi, INDIA

## Abstract

Although application of Zn combined with macronutrients (K, P, and N) can be used to fortify wheat grain with Zn, little is known about their interactions when foliar application is employed or the influences of common soil fertility management practices (e.g. N and straw management) on their efficiency. Therefore, the effects of foliar-applied Zn and N, P, or K on grain nutritional quality (especially Zn) were investigated in wheat grown under different soil N rates at two sites with (Sanyuan) or without (Yangling) employing straw return. A 4-year-long field experiment was also conducted to evaluate the environmental stability of the foliar formulations. Across 6 site-years, foliar Zn application alone or combined with N, P, or K fertilizers resulted in 95.7%, 101%, 67.9% and 121% increases in grain Zn concentration, respectively. In terms of increasing grain Zn concentration, foliar-applied Zn positively interacted with N (at Sanyuan) and K (at Yangling), but negatively interacted with P at any condition tested, suggesting depressive effects of foliarly-applied P on physiological availability of Zn. Although these interaction effects were the major factor that governing the efficiency of foliar-applied Zn combined with N, P, or K on grain Zn concentration, the magnitude of the increase/decrease in grain Zn (–3.96~5.71 mg kg^-1^) due to these interactions was much less than the average increases following Zn+K (31.3), Zn+P (18.7), and Zn+N (26.5 mg kg^-1^) treatments relative to that observed in foliar Zn-only treatment. The combined foliar application of Zn with N, P, or K did not cause any adverse impact on grain yield and other nutritional quality and in some cases slightly increased grain yield and macronutrient concentrations. Grain phytic acid:Zn molar ratios were respectively 52.0%, 53.1%, 43.4% and 63.5% lower in the foliar Zn, Zn+N, Zn+P and Zn+K treatments than in the control treatment. These effects were consistent over four years and across three soil N rates. Overall, combined foliar application of Zn with N, P, or K can successfully fortify wheat grain with Zn (above 40 mg kg^-1^), and including Zn in foliar N or K application are preferred for practically increasing dietary Zn intake.

## Introduction

Zinc deficiency in humans is a well-documented problem that can cause impairments in brain function, the immune system, and growth [1]. Inadequate dietary Zn intake has been identified as the major reason behind this global problem, particularly in the majority of people in developing countries, whose diets are dominated by wheat [2]. Thus, increasing Zn concentrations in wheat grain using agricultural tools such as Zn fertilization is receiving increased attention to effectively combat health problems related to Zn deficiency [3, 4].

Zinc fertilization methods include soil and foliar applications, the use of Zn-enriched NPK fertilizers, and seed priming [5, 6]. Numerous studies have demonstrated marked increases in grain Zn concentrations due to foliar Zn spray, whereas soil Zn applications and seed priming are less effective [6, 7, 8]. Recently, scientists and the HarvestPlus program (www.harvestplus.org) have emphasized the use of Zn in combination with foliar N, P, and K fertilizers to practically combat Zn deficiency [5]. Given that the root absorption capacity of Zn and macronutrients (e.g., N, P, and K) is easily compromised by drought or salinity, particularly during the grain-filling stage [9], the simultaneous and effective delivery of these nutrients through foliar applications is of great importance from an economic, agronomic and environmental point of view. However, the current understanding of possible factors affecting the efficiency of these fertilizers is incomplete.

It is well known that interactions between nutrients should be considered during fertilization [10]. However, to our knowledge, the effects of the interaction of foliar-applied Zn with K, P, or N on grain Zn accumulation under field conditions have not been investigated. Particularly, information on the role of K fertilization in Zn accumulation in wheat is lacking, although several experiments have indicated a substantial increase in Zn-chelation compounds (e.g., amino acids) and protein synthesis owing to K fertilization [11], as well as a favorable effect of K fertilization on Zn translocation in jute and a distinct alleviation of Zn deficiency in corn [12, 13]. These observations point to the likely involvement of K in the accumulation of Zn in plants, suggesting the necessity of further investigations on Zn biofortification of wheat.

So far, most of the studies assessing the relationship between P or N fertilizers and Zn accumulation in grains have been carried out through soil fertilization rather than foliar applications. For example, soil P fertilization inhibits the root uptake of Zn by altering soil factors (e.g., pH) and decreasing mycorrhizal colonization and phytosiderophore production [14, 15, 16]. In contrast, high soil N supply has been demonstrated to increase root exudation of phytosiderophores (nicotianamine, deoxymuigenic acid, avenic acid etc.) and expression of root Zn transporters (Zinc-, Iron-Permease family/ZRT-, IRT- like proteins), which could favor the mobility and root uptake of soil-applied Zn [2, 17]. These studies indicate direct influences of P and N on Zn in soil or root tissue, but do not clarify whether these nutrients will interact through foliar applications. Recent studies indicated that Zn translocation from root to shoot or from leaves to grains is not limited by high soil P applications [16, 18], indicating low impacts of P on the physiological availability of foliar-applied Zn in wheat plants. Thus, simultaneous foliar applications of Zn and P might be useful to facilitate the delivery of both P and Zn to crop tissues, which remains to be addressed. Urea has a greater influence on protein accumulation than pre-anthesis soil N applications under field conditions [19]. Together with the fact that urea can also act as a penetration enhancer [20] and stimulate the cuticular penetration of leaf-sprayed micronutrient in different plants [21], it can be speculated foliar-applied Zn may more effectively enhance grain Zn accumulation when it is applied concurrently with foliar N (urea) applications. Another aspect that remains poorly investigated under field conditions is the impact of foliar-applied Zn on wheat when combined with foliar N, P, and K fertilizers. Therefore, there is an urgent need to understand the complex interactions between foliar-applied Zn and N, P, and K fertilizers to ensure the efficient use of Zn-containing N, P, and K fertilizers.

The efficacy of foliar fertilizers might also be governed by other factors such as environmental conditions and common soil fertility management practices [10]. These factors may interact to alter the absorption and translocation of foliar-applied nutrients [22]. In China, excessive N fertilizer is commonly applied by farmers in wheat production (around 150–300 kg N ha^-1^), which decreased Chinese N fertilizer recovery efficiency by 13% relative to the world average (about 33%), and caused severe environmental problems [23]. Currently, Chinese farmers have encouraged to reduce soil N input and adopted straw incorporation to increase soil organic concentrations and nutrient contents, as well as alleviate environmental pollution [24]. Although it seems obvious that the soil nutrient concentrations caused by straw incorporation under different soil N application rates can be different (especially soil N and K concentration), little is known about how the efficiency of Zn combined with macronutrient fertilization is affected by straw and N management, and few studies have evaluated their applicability under varying environmental conditions.

Therefore, a 4-year-long field experiment was conducted in two locations to: (i) evaluate the efficacy of foliar-applied Zn combined with N, P, or K under varying environmental conditions and straw/soil N management strategies; (ii) quantify the effects of interactions between foliar-applied Zn and N, P, or K fertilizers on grain Zn, K, P, or N concentrations.

## Material and methods

### Site description

We confirm that the owner of the land gave permission to conduct the study on this site. Also, our field studies did not involve endangered or protected species. Field experiments were conducted in two experimental farms of the Northwest A&F University located in Yangling (108°04′E, 34°17′N; 524.7 m above sea level) and Sanyuan (108°52′E, 34°36′N; 427.4 m above sea level), in Shaanxi Province, China. The experiments were carried out from 2010 to 2014 in Yangling and from 2012 to 2014 in Sanyuan during the winter wheat (*Triticum aestivum* L.) growing season (October–June). The wheat cultivars used were Xiaoyan-22 at Yangling and Mianyang-26 at Sanyuan. The average annual rainfall is 600 mm in Yangling and 527 mm in Sanyuan. The climate in both regions is semi-humid with an average annual air temperature of 13.0°C in Yangling and 12.9°C in Sanyuan.

The soil properties at the experimental sites were different, especially the soil organic matter and available K concentrations, which were both much higher at Sanyuan than at Yangling (Table 1). These differences were largely due to the different straw management practices at the two locations. At Sanyuan, all the wheat straw had been chopped and returned to the field since 2008, whereas at Yangling, the wheat and maize straw was not returned. The soil available P concentration was also higher at Sanyuan than at Yangling (Table 1). All the soils were calcareous with high pH values (Table 1).

**Table 1:** Selected soil properties of the experimental sites at Yangling and Sanyuan.

Location	DTPA-Zn (mg kg^-1^)	DTPA-Fe (mg kg^-1^)	NO_3_^–^-N (mg kg^-1^)	NH_4_^+^-N (mg kg^-1^)	Available P (mg kg^-1^)	Available K (mg kg^-1^)	OM (g kg^-1^)	pH
**Yangling**	0.65	4.80	3.03	11.4	10.9	150	15.7	8.2
**Sanyuan**	0.90	2.96	3.80	12.7	13.3	209	23.4	8.3

Note:

OM: Organic matter

### Experimental design

The experiment was a split plot design with four replications in each of the two locations. The three main plot treatments had different soil N application levels, which were part of on-going long-term experiments, and the site had been treated with the same three fertilizer treatments every year since 2002 at Yangling and 2008 at Sanyuan. The three soil N application levels used at Yangling were as follows: no N application (N1, 0 kg N ha^-1^), 50% of the conventional N application (N2, 120 kg N ha^-1^), and the conventional N application used by local farmers (N3, 240 kg N ha^-1^). At Sanyuan, the three soil N application levels were as follows: 70% of conventional N application (recorded as N1 105 kg N ha^-1^), 85% of conventional N application (recorded as N2, 127.5 kg N ha^-1^), and conventional N application used by local farmers (recorded as N3, 150 kg N ha^-1^). A winter wheat-summer fallow rotation system was employed at Yangling, and a winter wheat-summer maize rotation system was employed at Sanyuan. The main plot size was 6 × 9.9 m at Yangling and 12 × 59 m at Sanyuan. Urea was used as the N source. Wheat was sown in mid-October and harvested in early June of the following year. Phosphorus was applied during planting as superphosphate at a rate of 100 kg P_2_O_5_ ha^-1^ at Yangling and 110 kg P_2_O_5_ ha^-1^ at Sanyuan. The soil N and P fertilizers were spread uniformly across the soil surface and then incorporated into the soil (approximately 15–20 cm) by plowing at sowing.

In 2010–2011 and 2011–2012, the following subplot treatments were applied to the plots at Yangling: CK (foliar application of deionized water), Zn (foliar application of 0.3% ZnSO_4_ 7H_2_O), Zn+P (foliar application of 0.3% ZnSO_4_·7H_2_O plus 0.2% KH_2_PO_4_ solution), and Zn+N (foliar application of 0.3% ZnSO_4_·7H_2_O plus 1.7% urea solution). In 2012–2013 and 2013–2014, the following subplot treatments were applied to the plots at both locations: CK, Zn, Zn+K (foliar application of 0.3% ZnSO_4_·7H_2_O plus 0.5% K_2_SO_4_ solution), Zn+P, and Zn+N. To separate the effects of the K, P, and N fertilizers, foliar N fertilization (1.7% urea solution) in 2012–2013, and foliar K fertilization (0.5% K_2_SO_4_ solution) and foliar P fertilization (0.2% KH_2_PO_4_ solution) in 2013–2014 were included at both locations. The sub-plot size was 2 × 1 m at both locations. All the foliar fertilizer treatments were applied three times (at 7-d intervals) during the early milk stage (April 29-May 13) using a hand sprayer (Youhua Plastic Co., Ltd., Taizhou city, Zhejiang Province, China) and a volume of 1000 L ha^-1^. All the solutions included 0.02% (v/v) Tween 20 as a surfactant.

### Measurements and statistical analyses

Grains were harvested at full maturity to determine the grain yield and grain concentrations of Zn, K, P, N, phytic acid, and Fe. The samples used for micronutrient (Zn, Fe) analyses were first dry-ashed at 550°C for 6 h, and then dissolved with 50% HNO_3_ (v/v) [25]. The micronutrient concentrations in the resulting solutions were analyzed using atomic absorption spectroscopy (AA320CRT, Shanghai, China). Additional samples were digested with concentrated H_2_SO_4_-H_2_O_2_[26]. Potassium concentrations were then determined using flame photometer, P concentrations were estimated by vanadate–molybdate–yellow colorimetry [26], and N concentrations were determined using the Kjeldahl method[27]. Grain phytic acid (PA) concentrations were determined using ion exchange resin chromatography to qualitatively estimate the bioavailability of Zn as the PA/Zn ratio in grain [28].

All data were analyzed using DPS version 7.05 statistical software (DPS) (Ruifeng Information Technology Co., Ltd., Hangzhou, China). To study the efficacy of foliar-applied Zn combined with N, P, or K under various soil N supplies, analysis of variance (ANOVA) of the main effects (soil-applied N and foliar fertilizer—including control, Zn, Zn+N, Zn+P and Zn+K treatments) was determined using the general linear models (GLM) procedure. To study the interaction effects of foliar-applied Zn with N, P, or K, the data were analyzed with two-factor ANOVA—factors in the N–Zn interaction analysis were foliar-applied Zn and foliar-applied N (including control, N, Zn, and Zn+N treatments); factors in the P–Zn interaction analysis were foliar-applied Zn and foliar-applied P (including control, P, Zn, and Zn+P treatments); and factors in the K–Zn interaction analysis were foliar-applied Zn and foliar-applied K (including control, K, Zn, and Zn+K treatments). Significant differences between means were determined by Fisher’s protected least significant difference (LSD) test at 95% confidence.

## Results

### Grain Zn concentration

When averaged across cropping season, foliar Zn application significantly increased the grain Zn concentration by 107% at Yangling and 78.3% at Sanyuan compared with the control treatment (Table 2). The increase in the grain Zn concentration caused by the Zn+N treatment was similar to that caused by the foliar Zn treatment for all soil N rates at Yangling. However, the increase in grain Zn was 9.0% higher in the foliar Zn+N treatment than in the foliar Zn treatment at Sanyuan. The addition of P to foliar-applied Zn significantly reduced grain Zn concentrations by an average of 14.6% at Yangling and 13.7% at Sanyuan compared with that following the Zn-only treatment. The increase in the grain Zn concentration under the Zn+K treatment was 29.7% higher than that observed under the Zn-only treatment at Yangling, but similar to that of the Zn-only treatment at Sanyuan. The grain Zn concentration at both locations also increased with increasing soil N rates (Table 2), and the increase was significant under the control treatment in 2011–2012 at Yangling, and under the control, foliar Zn, Zn+N and Zn+K treatments in 2013–2014 at Sanyuan (Table 2). Generally, the effects of the foliar fertilizers and soil N rates were quite similar during the studied cropping seasons.

**Table 2:** Grain Zn concentration (mg kg^-1^) of winter wheat as influenced by soil N fertilizer rate and foliar fertilizer applications under field conditions at Yangling during 2010–2014 and Sanyuan during 2012–2014.

Treatment^b^	Yangling						Sanyuan		
	N1	N2	N3	N1	N2	N3	N1	N2	N3
	2010–2011			2011–2012			2012–2013		
**CK**	22.6e	23.0e	24.8e	21.3e	23.2de	29.3d	23.2g	29.4fg	33.0ef
**Foliar Zn**	40.1b-d	43.8ab	44.5ab	45.8a	45.4a	45.2a	49.3d	57.3bc	64.8a
**Foliar Zn+N**	42.0a-c	47.0a	42.7a-c	44.6ab	44.0a-c	42.4a-c	59.8ab	62.3ab	64.2a
**Foliar Zn+P**	34.3d	34.9d	36.9cd	37.3c	42.0a-c	37.9bc	39.5e	50.8cd	52.7cd
**Foliar Zn+K**	-^a^	-	-	-	-	-	51.8cd	55.8b-d	55.7b-d
	2012–2013			2013–2014			2013–2014		
**CK**	22.0g	24.5g	26.3g	23.5g	27.5f	27.7f	30.9g	34.0fg	36.5f
**Foliar Zn**	56.2de	56.0de	62.3a-c	55.2cd	57.4c	62.0b	48.0de	55.2bc	55.6bc
**Foliar Zn+N**	58.6cd	59.4b-d	60.0b-d	55.7cd	57.2c	56.7c	54.3bc	63.1a	56.4b
**Foliar Zn+P**	52.3ef	47.7f	49.9f	49.4e	50.3e	52.4de	45.7e	46.6e	49.2de
**Foliar Zn+K**	64.9ab	66.6a	66.9a	64.0ab	66.4a	66.2a	47.4e	54.4bc	52.0cd

Note:

^a^not measured

^b^Mean values followed by different lowercase letters are significantly different among combined soil N and foliar application treatments (P≤0.05).

As shown in Table 3, foliar N application resulted in an 8.6% increase in grain Zn concentration when foliar Zn was concurrently applied at Sanyuan, but the same was not true when Zn was not applied. Therefore, the increase in grain Zn concentration following foliar Zn+N application resulted from the significant positive interaction between foliar-applied Zn and N. However, there was no significant interaction between foliar-applied Zn and N at Sanyuan. The grain Zn concentration significantly decreased by an average of 8.9% owing to foliar P application in the absence of Zn and by an average of 13.1% when foliar Zn was applied. Therefore, a significant negative two-way interaction between foliar-applied Zn and P was observed at both locations, resulting in decreased grain Zn concentration. Moreover, at Yangling, the grain Zn concentration significantly increased when foliar K was applied with Zn, but the same was not true when K was applied alone. These results indicate significant positive interaction between foliar-applied Zn and K, resulting in increased grain Zn concentrations in the foliar Zn+K treatment. However, at Sanyuan, the foliar K application did not influence the grain Zn concentration in the treatment with or without concurrent foliar Zn application, and thus a significant interaction between foliar-applied Zn and K did not exist.

**Table 3:** Interactions between foliar-applied Zn and K, P, or N on Zn, K, P, or N concentrations in wheat grain.

**Location**	**Value**	**No foliar Zn**		**Foliar Zn**		**Zn×Ninteraction value**^a^	***F* value probabilities**		
		**-N**	**+N**	**-N**	**+N**		**Foliar Zn (a)**	**Foliar N (b)**	**a×b**
**Yangling**	**Yield (t ha**^**-1**^**)**	1.62c	1.68c	1.88b	2.01a	0.08	0.082	0.234	0.400
	**Zn concentration (mg kg**^**-1**^**)**	24.3b	22.7b	58.2a	59.3a	2.77	<0.001	0.229	0.662
	**N concentration (g kg**^**-1**^**)**	22.5d	24.2b	23.0c	24.8a	0.11	0.063	0.020	0.720
**Sanyuan**	**Yield (t ha**^**-1**^**)**	3.18a	3.14a	3.11a	3.21a	0.15	0.976	0.746	0.180
	**Zn concentration (mg kg**^**-1**^**)**	28.6 c	29.0 c	57.2 b	62.1 a	4.53*	<0.001	0.012	0.027
	**N concentration (g kg**^**-1**^**)**	20.7b	23.0a	19.7b	22.7a	0.34	0.148	0.041	0.594
		**No foliar Zn**		**Foliar Zn**		**Zn×P interaction value**	***F* value probabilities**		
		**-P**	**+P**	**-P**	**+P**		**Foliar Zn (a)**	**Foliar P (b)**	**a×b**
**Yangling**	**Yield (t ha**^**-1**^**)**	3.23a	3.19a	3.22a	3.39a	0.22	0.548	0.654	0.191
	**Zn concentration (mg kg**^**-1**^**)**	26.2c	23.0d	58.2a	50.7b	-4.27**	<0.001	<0.001	0.001
	**P concentration (g kg**^**-1**^**)**	1.90a	1.97a	2.03a	2.08a	-0.02	0.065	0.133	0.844
**Sanyuan**	**Yield (t ha**^**-1**^**)**	5.40b	5.33b	5.59ab	5.82a	0.31	0.273	0.699	0.125
	**Zn concentration (mg kg**^**-1**^**)**	34.1c	31.9c	52.9a	45.9b	-4.68*	<0.001	0.002	0.068
	**P concentration (g kg**^**-1**^**)**	2.78ab	2.94a	2.63b	2.87a	0.07	0.182	0.103	0.667
		**No foliar Zn**		**Foliar Zn**		**Zn×K interaction value**	***F* value probabilities**		
		**-K**	**+K**	**-K**	**+K**		**Foliar Zn (a)**	**Foliar K (b)**	**a×b**
**Yangling**	**Yield (t ha**^**-1**^**)**	3.23c	3.41b	3.22c	3.59a	0.20	0.553	0.217	0.115
	**Zn concentration (mg kg**^**-1**^**)**	26.2c	25.4c	58.2b	65.5a	8.14***	<0.001	<0.001	<0.001
	**K concentration (g kg**^**-1**^**)**	3.68b	3.63b	3.71 b	3.96a	0.25*	0.374	0.651	0.019
**Sanyuan**	**Yield (t ha**^**-1**^**)**	5.46c	5.78ab	5.59bc	5.98a	0.07	0.140	0.064	0.602
	**Zn concentration (mg kg**^**-1**^**)**	34.1b	31.7c	52.9a	51.5a	1.06	<0.001	0.843	0.124
	**K concentration (g kg**^**-1**^**)**	4.16c	4.31b	4.31b	4.57a	0.02	0.036	0.035	0.862

Note:

^a^The interactions were quantified as follows:

FoliarZn×FoliarK=[(FoliarZnK+CK)−(FoliarZn+FolairK)]/2

FoliarZn×FoliarP=[(FoliarZnP+CK)−(FoliarZn+FoliarP)]/2

FoliarZn×FoliarN=[(FoliarZnN+CK)−(FoliarZn+FoliarN)]/2

*, **, *** means significant difference at P≤0.05, P≤0.01 or P≤0.001 for F-test, respectively.

### Grain yield

At Yangling, grain yield was 36.7% higher in the N2 and N3 treatments than in the N1 treatment during all cropping seasons, whereas there was no significant difference between N2 and N3 during any cropping season, with the exception of 2010–2011 (Table 4). At Sanyuan, grain yield in the N2 treatment was 19.7% lower than in the N1 treatment in 2012–2013, while grain yield in the N3 treatment was 10.8% and 6.7% lower in the N1 treatment in 2012–2013 and 2013–2014, respectively. There was no significant two-way interaction between soil N and foliar application at both locations, but foliar Zn, Zn+N, Zn+P and Zn+K applications significantly increased grain yield at Yangling during 2012–2013 and at Sanyuan during 2013–2014 compared with the control treatment (Table 4). Furthermore, as shown in Table 3, significant two-way interactions between foliar-applied Zn and N, P or K did not exist at either location.

**Table 4:** Grain yield and protein concentration of winter wheat as influenced by soil N fertilizer rate and foliar fertilizer applications under field conditions at Yangling during 2010–2014 and Sanyuan during 2012–2014.

Treatment^b^	Yield (t ha^-1^)						Protein concentration (g kg^-1^)					
	Yangling				Sanyuan		Yangling				Sanyuan	
	2010–2011	2011–2012	2012–2013	2013–2014	2012–2013	2012–2013	2010–2011	2011–2012	2012–2013	2013–2014	2012–2013	2012–2013
**Soil N (kg ha**^**-1**^**)**												
**N1**	1.83C	3.93B	1.81A	2.78B	3.51A	5.89A	76B	97C	130B	109B	118B	99A
**N2**	3.48A	5.22A	1.83A	3.75A	2.82C	5.95A	111A	109B	131B	124A	120B	101A
**N3**	3.16B	5.12A	1.82A	3.64A	3.13B	5.49B	109A	118A	135A	128A	126A	102A
**Foliar application**												
**CK**	2.83A	4.71A	1.62C	3.23AB	3.18A	5.40C	93B	108AB	130B	118BC	119BC	99B
**Foliar Zn**	2.85A	4.78A	1.88B	3.22B	3.11A	5.59AB	96AB	106B	131B	119B	117C	97B
**Foliar Zn+N**	2.83A	4.80A	2.01A	3.52AB	3.21A	6.09A	107A	111A	140A	127A	128A	105A
**Foliar Zn+P**	2.79A	4.74A	1.79B	3.39AB	3.24A	5.82AB	99AB	107B	129B	113C	121BC	102AB
**Foliar Zn+K**	-^a^	-	1.80B	3.59A	3.03A	5.98A			131B	125A	122B	102AB

Note:

^a^not measured

^b^Mean values followed by different capital letters are significantly different among soil N or foliar application treatments (P≤0.05).

### Grain protein, P and K concentrations

When averaged across cropping season, grain protein concentration was 15.3% greater in the N2 treatment than in the N1 treatment at Yangling; further increasing the soil N rate to N3 increased grain protein concentration by 8.3% in 2011–2012 and 3.1% in 2012–2013 at Yangling, and by 5.0% in 2012–2013 at Sanyuan (Table 4). Foliar Zn+N application also resulted in 8.0% and 6.9% increases in grain protein concentrations when compared to the control treatment at Yangling and Sanyuan, respectively. As shown in Table 3, an insignificant interaction between foliar N and Zn was observed at either location, resulting similar increase in grain protein concentration following foliar N application with or without concurrent foliar Zn application.

Grain P concentration was 10.5% lower in the N2 treatment than in the N1 treatment in 2011–2012 at Yangling, and 6.3% lower in the N2 treatment than in the N1 treatment in 2012–2013 at Sanyuan (Table 5). However, at both locations, grain P concentration was significantly lower in the N3 treatment than in the N1 treatment in all cropping seasons with the exception of 2012–2013. Comparing with the control treatment, foliar Zn, Zn+N and Zn+K applications did not influence grain P concentration at Yangling, while foliar Zn+P application increased grain P concentration by 10.6% in 2013–2014. At Sanyuan, grain P concentration was increased by 10.6% due to foliar application of Zn+N in 2012–2013, and increased by 10.0% and 13.4% due to foliar Zn+P and Zn+K in 2013–2014, respectively. However, there was no significant influence of the interaction between foliar-applied Zn and P on grain P concentrations at either location (Table 3).

**Table 5:** Grain P and K concentration of winter wheat as influenced by soil N fertilizer rate and foliar fertilizer applications under field conditions at Yangling during 2010–2014 and Sanyuan during 2012–2014.

Treatment^b^	P concentration (g kg^-1^)						K concentration (g kg^-1^)					
	**Yangling**				**Sanyuan**		**Yangling**				**Sanyuan**	
	2010–2011	2011–2012	2012–2013	2013–2014	2012–2013	2012–2013	2010–2011	2011–2012	2012–2013	2013–2014	2012–2013	2012–2013
**Soil N (kg ha**^**-1**^**)**												
**N1**	3.49A	3.71A	2.72A	2.24A	2.56A	2.74A	4.49A	4.10A	3.74A	3.72A	4.20B	4.48A
**N2**	3.28A	3.32B	2.49A	2.04AB	2.40B	2.74A	4.49A	3.93AB	3.66A	3.61A	4.62A	4.31A
**N3**	2.89B	3.21B	2.77A	2.01B	2.43B	2.52B	4.01B	3.70B	3.71A	3.78A	4.30B	4.36A
**Foliar application**												
**CK**	3.41A	3.39A	2.65AB	1.99B	2.35B	2.61B	4.53A	3.77A	3.69A	3.61B	4.14B	4.17B
**Foliar Zn**	3.13A	3.47A	2.51B	2.12AB	2.34B	2.54B	4.19A	4.08A	3.63A	3.67B	4.30AB	4.38AB
**Foliar Zn+N**	3.11A	3.40A	2.52B	2.08AB	2.69A	2.36B	4.36A	3.94A	3.62A	3.73AB	4.39AB	4.40AB
**Foliar Zn+P**	3.23A	3.37A	2.72AB	2.20A	2.53AB	2.87A	4.25A	3.86A	3.73A	3.66B	4.45A	4.47A
**Foliar Zn+K**	-^a^	-	2.90A	2.09AB	2.41B	2.96A	-	-	3.84A	3.86A	4.58A	4.50A

Note:

^a^not measured

^b^Mean values followed by different capital letters are significantly different among soil N or foliar application treatments (P≤0.05).

Grain K concentration was significantly lower in the N3 treatment than in the N1 and N2 treatments at Yangling in 2010–2011, and higher in the N2 treatment than in the N1 and N3 treatments at Sanyuan in 2012–2013 (Table 5). However, there was no significant influence of soil N rates in 2011–2012 and 2013–2014 at either location. As shown in Table 3, the grain K concentration was not affected by foliar K application in the absence of Zn, but increased by 6.7% following the Zn+K treatment, resulting in a significant foliar K–Zn interaction. At Sanyuan, a significant interaction between foliar K and Zn did not occur, but foliar Zn+K resulted in 9.3% increase in grain K concentration compared to the control treatment.

### Grain PA concentration

**Fig 1:**
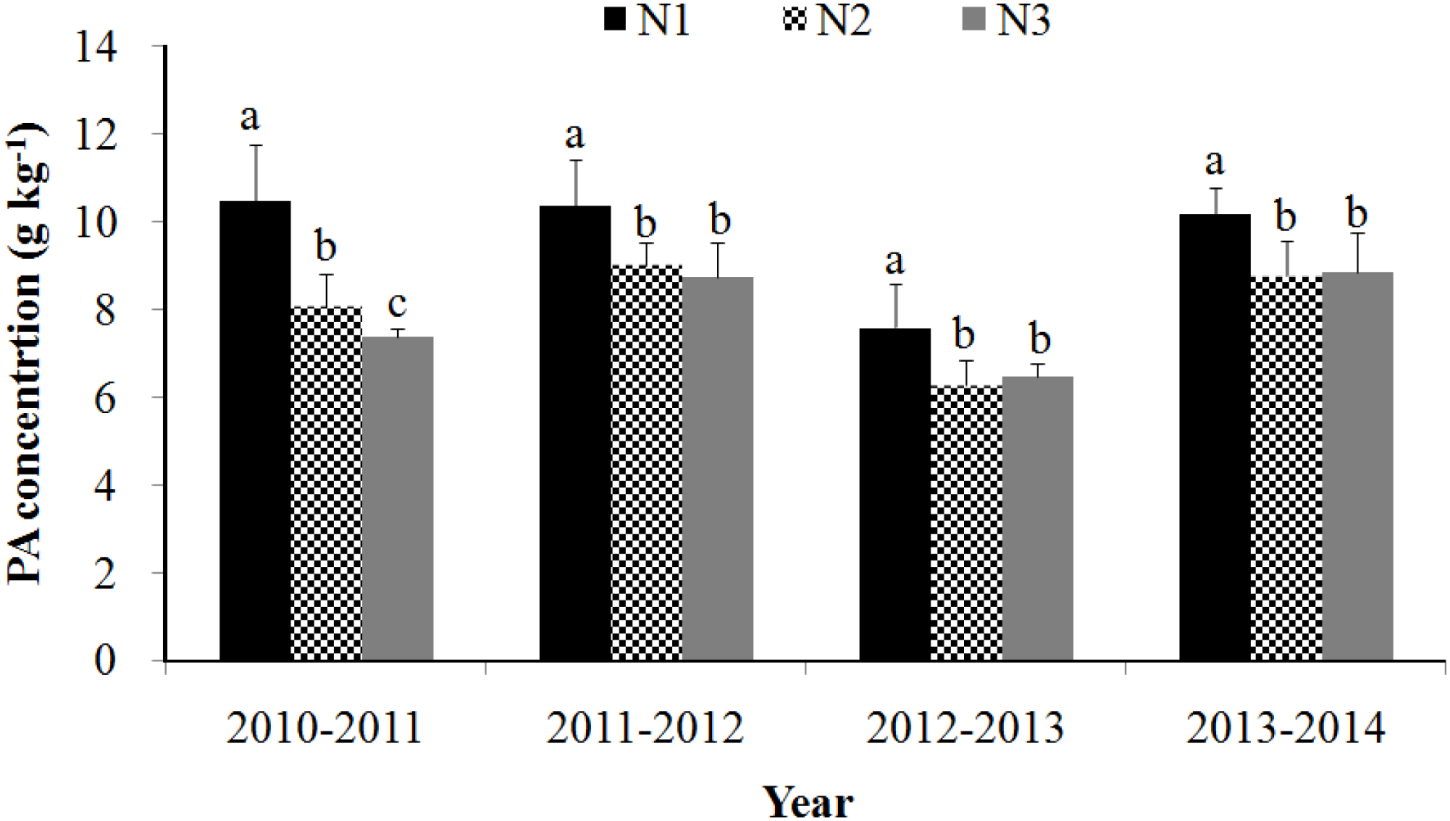
Grain PA concentration of winter wheat as influenced by soil N fertilizer rate (0 [N1], 120 [N2] or 240 [N3] kg N ha^-1^) under field conditions at Yangling during 2010–2011, 2011–2012, 2012–2013 and 2013–2014. PA: phytic acid. Error bars represent standard error (n = 20). Bars having different lowercase letters are significantly different (*P* < 0.05). Because the effect of the foliar application was not significant for grain PA concentration, the mean grain PA of different foliar treatments for each cropping seasons and all the N rates are shown.

Grain PA concentration was only significantly affected by the main effect of soil N in all cropping seasons (Fig 1). When averaged across the cropping seasons, increasing soil N application rate from N1 to N2 or N3 resulted in 16.9% and 23.5% decrease in grain PA concentration, respectively. However, there was no significant difference between N2 and N3 in all cropping seasons with the exception of 2010–2011, where grain PA concentration was significantly lower in the N3 than in the N2 treatment. Moreover, grain PA concentration was not affected by foliar fertilizer application in all cropping seasons.

### Grain Zn bioavailability

**Fig 2:**
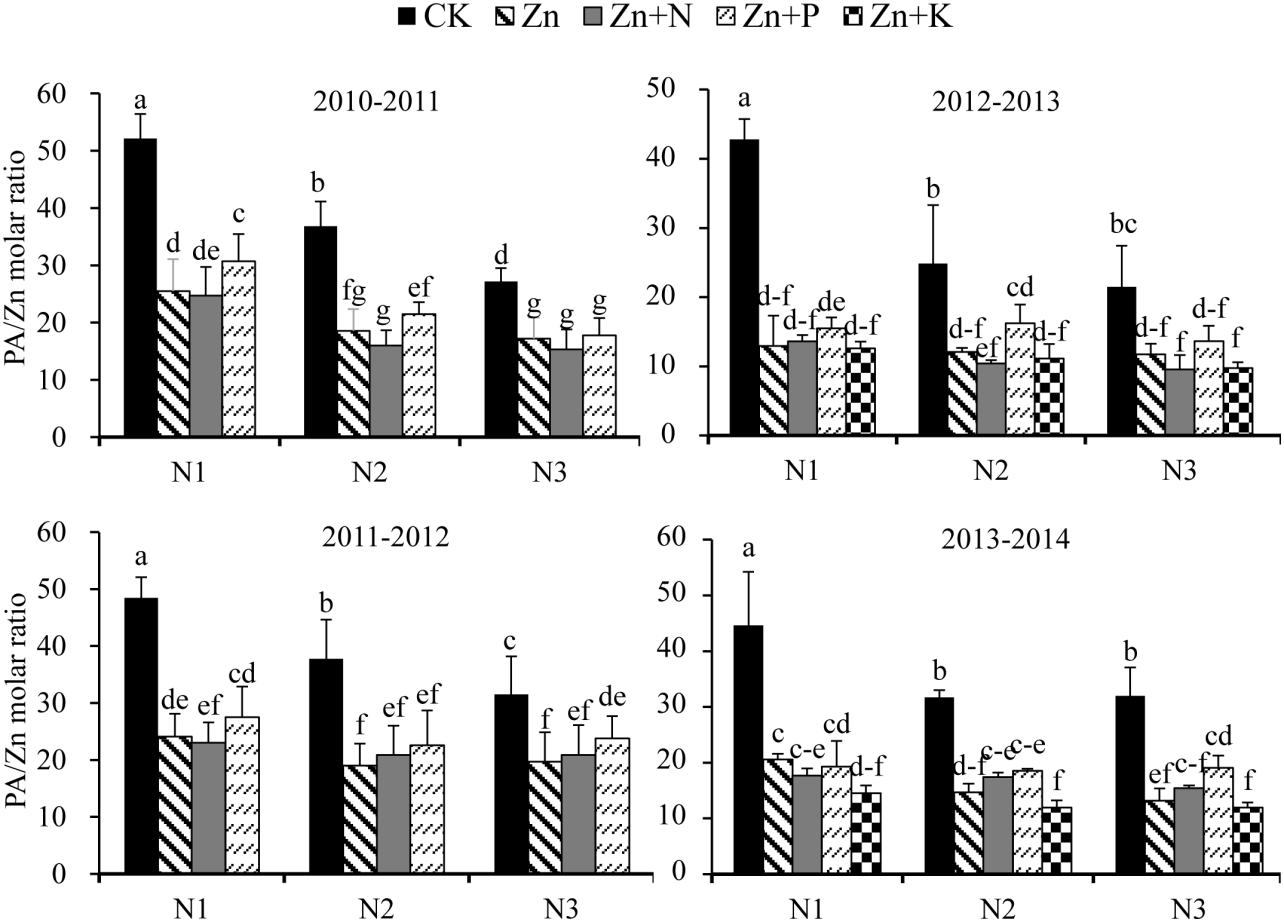
Grain PA/Zn molar ratio of winter wheat as influenced by soil N fertilizer rate (0 [N1], 120 [N2] or 240 [N3] kg N ha^-1^) and foliar fertilizer applications (distilled water spray [CK], ZnSO_4_·7H_2_O [Zn], ZnSO_4_·7H_2_O+urea [Zn+N], ZnSO_4_·7H_2_O+KH_2_PO_4_ [Zn+P], and ZnSO_4_·7H_2_O+ K_2_SO_4_ [Zn+K]) at Yangling. In 2010–2011 and 2011–2012, foliar Zn+K were not applied. Error bars represent standard error (n = 4). PA: phytic acid. Bars having different lowercase letters are significantly different (*P* < 0.05).

The effects of soil-applied N, foliar fertilizer as well as their interaction on grain PA/Zn molar ratio were significant (Fig 2). Grain PA/Zn was reduced by an average of 52.0, 53.1, 43.4 and 63.5% with the foliar application of Zn, Zn+N, Zn+P and Zn+K, respectively. Also, grain PA/Zn molar ratio was generally higher in the foliar Zn+P treatment than in the foliar Zn-only treatment, but lower in the foliar Zn+K treatment. Grain PA/Zn molar ratio was also reduced by an average of 30.6% and 40.3% under N2 and N3, respectively, in the absence of foliar Zn application. However, there were no significant difference in grain PA/Zn molar ratio among soil N rates in the presence of foliar Zn, Zn+N, Zn+P and Zn+K applications during all cropping seasons with the exception of 2010–2011, where grain PA/Zn was significantly lower in the N2 and N3 than in the N1 treatment.

## Discussions

The chemical form and water solubility of fertilizer formulations can affect the agronomic effectiveness of foliar fertilizers [22]. The macronutrient fertilizers used in the present study (K_2_SO_4_, KH_2_PO_4_, and urea) are highly soluble. Because K^+^, urea, and H_2_PO_4_^-^ in fertilizer solutions have a relatively small molecular size and weight, they are easily absorbed by crops, and thereby widely applied by farmers to correct macronutrient deficiency during the late growth period of wheat. In this study, the inclusion of ZnSO_4_ (Zn^2+^) in foliar macronutrient fertilizers did not produce any visible precipitation, indicating the applicability of these foliar formulations with respect to their physico-chemical properties. Moreover, our previous study [29] conducted at one location with no straw returning showed that the effectiveness of foliar Zn application in increasing grain Zn could be weakened by combined foliar P application, but not affected by foliar N application. In the present study, we further clarified the effectiveness of combined foliar Zn and K application in enhancing grain Zn concentration, and quantified the effects of interactions between foliar-applied Zn and N, P, or K fertilizers. Also, the efficacy of foliar-applied Zn combined with N, P, or K under varying environmental conditions and locations with different straw/soil N management strategies were evaluated in the present study. The results showed that the targeted levels of Zn (40~45 mg kg^-1^) in grain for improved human nutrition can be easily reached by simultaneous foliar applications of Zn combined with N, P, or K irrespective of environmental conditions, or N and straw management. Furthermore, depending on location, positive interactions between foliar-applied Zn combined with N and K were observed. However, foliar-applied P negatively interacted with foliar-applied Zn at both locations. These results indicate that the interactions between Zn and macronutrients at the physiological level are important for determining the efficiency of various fertilizers.

To our knowledge, this is the first study that shows a synergistic effect of foliar-applied K and Zn on grain Zn concentrations. Foliar-applied Zn is be absorbed by the leaf epidermis through possible pathways such as stomata and trichomes [30]. Foliar-applied K possibly favors the penetration of Zn through the epidermis because the application of K stimulates stomatal opening [31]. Moreover, K fertilization has been reported to accelerate N uptake and its assimilation in mustard plants by improving the formation of amino acids required for protein synthesis [32]. The transport of amino acids can be enhanced through K fertilization [11]. These positive effects of K on N may, in turn, increase the absorption and translocation of foliar-applied Zn because of the important role of N in the chelation and transport of Zn, which has been previously reported [33, 34]. Consistent with this suggestion, a simultaneous increase in grain Zn and N concentrations due foliar Zn+K fertilization, and significant positive correlations between grain Zn and K concentrations were found at Yangling (Table 6). However, compared to the foliar Zn only treatment, the inclusion of K in the foliar Zn application resulted in similar increase in grain Zn at Sanyuan. According to those results, it is obvious that the positive interaction between foliar Zn and K is dependent on location, but there is no negative/competitive effect of K (K^+^) on the absorption and translocation of foliar-applied Zn.

**Table 6:** Correlation coefficients between nutrients concentrations in grain of winter wheat grown at different soil N fertilizer rates and foliar fertilizer applications at Yangling during 2010–2014 and Sanyuan during 2012–2014.

Items	**Yangling**				**Sanyuan**		
	Protein	P	K	PA	Protein	P	K
**Zn**	0.46**	-0.36*	-0.35*	-0.17	0.24	-0.03	0.38*
**Fe**	0.55**	-0.49**	-0.67**	-0.04	-0.68**	0.18	0.03
**Ca**	-0.18	0.22	0.53**	-0.36*	0.89**	-0.34	-0.05

Note:

PA: Phytic acid

*, **, *** means significant difference at P≤0.05, P≤0.01 or P≤0.001 for F-test, respectively.

Significant antagonistic effects of foliar P and Zn observed in the present study indicate negative effects of foliar-applied P on the physiological availability of Zn in vegetative tissues. However, this effect was different from that of root-applied P. Previous studies have shown that reductions in tissue Zn concentrations caused by high soil P supply are mediated primarily by mycorrhiza [18], whereas the transfer of Zn is not affected when P is applied in AM-free solution cultures [35, 18] or on non-mycorrhizal species such as canola [36]. Zhang et al. [16] reported that translocation of foliar-applied Zn from vegetative tissues into grain was not inhibited by high P supply in soil. These results suggest that the influence of P on Zn in vegetative tissue varies among different P fertilization methods, and the physiological availability of applied Zn in leaf tissue is more strongly inhibited by foliar-applied P than soil or root-applied P. As described in detail previously [29], foliar P application likely inhibits the transport of Zn from vegetative tissues to grains by decreasing the physiological availability of leaf Zn by reducing Zn solubility or increasing the Zn-binding properties of cell walls [37–39]. Although the concurrent application of foliar P and Zn decreased the grain Zn concentration by 14.9% relative to the foliar Zn treatment, there was a 95.3% increase in grain Zn due to the Zn+P treatment relative to the CK treatment, and this increase was markedly higher than the decrease in grain Zn caused by foliar P application (average 9.3%). Therefore, the adaptation of foliar Zn application is needed to restore the loss of Zn caused by foliar or soil P applications.

Foliar-applied N was as effective as soil-applied N in increasing grain Zn concentrations, which highlights the importance of foliar N application for grain Zn biofortification. However, the synergistic effect of foliar-applied Zn and N on grain Zn concentrations was more evident than that of soil N application at Sanyuan. This supports our previous assumption that the effectiveness of foliar-applied Zn is more beneficial when combined with foliar-applied N. The rapid absorption and assimilation of urea to amino acids [40] may increase the absorption and translocation of foliar-applied Zn because amino acids can improve Zn availability for plants by coordinating metal ions (such as Zn) via their carboxyl groups [41]. Also, there is a close genetic link between the remobilization of Zn and amino acids from leaf tissue to grains [42]. In the present study, foliar N application increased grain protein concentrations, which might be an important sink for Zn as reviewed by Cakmak et al. [2]. Thus, the synergistic relationship between foliar-applied Zn and N might be due to the higher grain Zn storage capacity of plants treated with foliar-applied N, which thereby induces Zn transportation from vegetative tissue into seeds [33]. Consistent with this suggestion, significant positive correlations between grain protein and Zn concentrations were found in the present study. Altogether, these observations suggest that foliar N application is an efficient approach to maximize Zn accumulation when foliar Zn application is adopted, whereas soil N application is insufficient.

In this study, the effect of foliar fertilizers on grain Zn concentrations was quite similar in different years, suggesting a low influence of environmental factors such as rainfall and temperature. However, there was considerable location-to-location variation in the Zn-N and Zn-K interactions, which might be partially due to the different wheat cultivar used in the two locations. As indicated by Gomez-Coronado et al. [43], different wheat cultivar varied in their capacity to accumulate zinc, and therefore respond differently to Zn applications. Moreover, the variation in basal nutrient concentration may also played a role. As presented in Table 1, due to the adoption of different straw management practices, the available soil K at Yangling was significantly lower than that at Sanyuan, which may have caused the higher response of grain Zn to foliar K application at Yangling. Straw return can also replenish the soil Zn reservoir as indicated by the higher soil Zn concentration resulting from straw incorporation in the present study. It has been shown previously that increasing soil N rate does not increase soil Zn concentration [44], but enhances root Zn uptake [45], possibly through the improvement of Zn availability in the rhizosphere by exudation of organic acids [46]. Also, the positive effect of N supply on Zn accumulation may be more evident when the Zn reservoir in soil or vegetative tissues is high [2, 33]. Thus, the higher impact of soil and foliar N on grain Zn concentration observed at Sanyuan in the present study may be partially due to replenishment of the Zn reservoir at Sanyuan caused by straw returning. However, N may not be the primary limiting factor for Zn accumulation at Yangling. Other nutritional constraints (such as K limitation) may have existed at Yangling that curtailed the response of grain Zn to foliar N application. In contrast, reductions in grain Zn concentration due to foliar P application did not differ between the two locations, indicating that a negative effect of foliar P application on wheat Zn occurred irrespective of wheat cultivar and basal nutrient concentrations. This further emphasizes the necessity of adopting Zn biofortification methods (e.g., foliar Zn application) to offset Zn loss. However, studies under diverse environmental conditions and on larger regional scales are needed to further test the applicability of foliar-applied Zn and macronutrients.

Phytate in staple foods can complex with Zn and reduce its absorption into the human body. The PA/Zn molar ratio is considered to be an indicator of nutritional quality [47]. A PA/Zn ratio of ≥ 15, 5–15, and < 5 is equal to an absorption rate of 20%–55%, 30%–35%, and 10%–15%, respectively [48]. In the present study, foliar fertilization treatments had little effect on grain PA concentrations, but distinctly decreased grain the PA/Zn molar ratio. This suggests the possibility of increasing the Zn nutritional quality of wheat grain through these agronomic approaches. Our results also indicate that the Zn+P treatment was less effective in decreasing the grain PA/Zn ratio than the Zn-only treatment, whereas the Zn+K and Zn+N treatments were more effective.

## Conclusions

Depending on location, foliar-applied Zn combined with K or N was equally or more effective in improving grain Zn concentrations than the Zn-only treatment. The Zn+P treatment was less effective in increasing grain Zn concentrations than the Zn-only treatment due to the antagonistic effects of P and Zn. The efficiency of the foliar fertilizers was not generally affected by cropping season, though this result was inconsistent among locations, indicating a strong impact of basal nutrient concentrations resulting from straw management (soil available K in particular). Furthermore, the combination of foliar Zn and foliar N, P, or K applications did not decrease, but in some cases slightly increased grain yield, N, P, and K concentrations. For the purpose of food and nutrient security, foliar N and K application is necessary to maximum the response of grain Zn concentration to foliar-applied Zn. Also, the integration of foliar-applied Zn with foliar P application is recommended to offset P-induced grain Zn reductions.
